# Cyclin D1 A870G polymorphism and the risk of colorectal cancer and adenoma

**DOI:** 10.1038/sj.bjc.6603007

**Published:** 2006-02-21

**Authors:** E S Schernhammer, G J Tranah, E Giovannucci, A T Chan, J Ma, G A Colditz, D J Hunter, W C Willett, C S Fuchs

**Affiliations:** 1 Channing Laboratory, Department of Medicine, Brigham and Women's Hospital and Harvard Medical School, 181 Longwood Avenue, Boston, MA 02115, USA; 2 Ludwig Boltzmann – Institute for Applied Cancer Research, KFJ-Spital, Vienna, Austria; 3 Applied Cancer Research – Institute for Translational Research Vienna (ACR – ITR VIEnna), Vienna, Austria; 4 Department of Epidemiology, Harvard School of Public Health, Boston, MA, USA; 5 Harvard Center for Cancer Prevention, Boston, MA, USA; 6 Department of Nutrition, Harvard School of Public Health, Boston, MA, USA; 7 Gastrointestinal Unit, Massachusetts General Hospital, Boston, MA, USA; 8 Epidemiology Program, Dana-Faber/Harvard Cancer Center, Boston, MA, USA; 9 Department of Adult Oncology, Dana-Farber Cancer Institute, Boston, MA, USA

**Keywords:** cyclin D, *CCND1*, A870D, polymorphism, colorectal cancer, colorectal adenoma

## Abstract

Cyclin D1 (*CCND1*) plays a key role in cell cycle control, particularly in the transition from G_1_ to S phase, which is regulated by cyclin-dependent kinases. A common adenine to guanine polymorphism (A870G) in the *CCND1* gene has been associated with a longer-life protein and an increased risk of colorectal cancer and adenoma in some studies. Among subjects with hereditary nonpolyposis colorectal cancer, the A870G polymorphism has also been associated with a younger age of onset of colorectal cancer. We analysed 181 colorectal cancer cases and 475 matched controls and 524 adenoma cases and 517 matched controls within women in the Nurses’ Health Study (NHS) cohort, 171 colorectal cancer cases and 347 matched controls and 372 adenoma cases and 712 matched controls nested within men in the Health Professionals’ Follow-Up Study (HPFS) cohort, and 258 colorectal cancer cases and 415 matched controls within men in the Physicians’ Health Study (PHS) cohort to assess the risk associated with the *CCND1* A870G genotype. Moreover, we assessed whether *CCND1* genotype modified the effect of a sporadic (nonsyndromic) family history of colorectal cancer as well as the effect of other dietary and lifestyle risk factors for colorectal cancer and adenoma. In all cohorts combined, the *CCND1* polymorphism did not show statistically significant associations to risk of colorectal cancer (odds ratio (OR) for A allele carriers, 1.04; 95% confidence interval (95% CI), 0.82–1.32) or adenoma (OR, 0.96; 95% CI, 0.79–1.18). The *CCND1* A870G genotype was associated with a modest, although nonsignificantly elevated risk of colorectal cancer (OR, 1.59; 95% CI, 0.98–2.57) in women. In contrast, the polymorphism was not associated with increased risk of adenoma in either men or women. Among participants with the A870G genotype, a family history of colorectal cancer conferred a substantially greater risk of colorectal cancer in the women (*P* for interaction=0.06) and adenoma in the men (*P* for interaction=0.02). Current postmenopausal hormone (PMH) use was associated with a significant reduction in the risk of colorectal cancer and adenoma among women with the A870G genotype, whereas there was no effect of PMH use among those with the GG genotype. The *CCND1* polymorphism appeared to confer a modest elevation in the risk of colorectal cancer among women. Moreover, the A870G genotype may enhance the protective effect of postmenopausal oestrogen use on the development of colorectal neoplasia.

Cyclin D1 (*CCND1*) is a protein that plays a key role in cell cycle control, particularly in the transition from G_1_ to S phase, which is regulated by cyclin-dependent kinases (Sherr, 1996). The overexpression of the *CCND1* gene occurs in more than one-third of all colorectal cancers (Arber *et al*, 1996) and adenoma (Weinstein *et al*, 1997), and expression of an antisense *CCND1* cDNA suppresses the growth of colon cancer cells in animal models (Arber *et al*, 1997).

*Cyclin D1* mRNA is alternatively spliced between exons 4 and 5 to generate two transcripts, which may be concurrently transcribed in various human tissues (Betticher *et al*, 1995). A common G to A polymorphism at codon 242 on exon 4 in the *CCND1* gene has been shown to modulate splicing of the *CCND1* transcript (Betticher *et al*, 1995). The dominant A allele preferentially generates the truncated transcript (Weinstein *et al*, 1997; Sawa *et al*, 1998), which encodes a cyclin D1 protein with a longer half-life. This accumulation of cyclin D1 in the cell may promote cell proliferation and the subsequent development of colorectal cancer.

Previous studies have had inconsistent results for an association of the *CCND1* A870G polymorphism with bladder cancer (Cortessis *et al*, 2003; Wang *et al*, 2003b; Berman *et al*, 2004; Ito *et al*, 2004), endometrial cancer (Kang *et al*, 2005), breast cancer (Grieu *et al*, 2003; Krippl *et al*, 2003; Shu *et al*, 2005), head and neck cancer (Zheng *et al*, 2001), gastric and oesophageal cancer (Wang *et al*, 2003c; Zhang *et al*, 2003a), hepatocellular carcinoma (Zhang *et al*, 2003b), lung cancer (Qiuling *et al*, 2003), and prostate cancer (Koike *et al*, 2003; Wang *et al*, 2003a). The strongest evidence, to date, has linked the *CCND1* A870G polymorphism with an increased risk of colorectal cancer and adenoma in many (Kong *et al*, 2000, 2001; Bala and Peltomaki, 2001; Porter *et al*, 2002) though not all studies (McKay *et al*, 2000). Moreover, the *CCND1* A870G genotype (Kong *et al*, 2000) or the presence of the variant truncated *CCND1* transcript (preferentially encoded by the A870G genotype) (Bala and Peltomaki, 2001) was associated with a younger age of cancer onset in subjects with hereditary nonpolyposis colorectal cancer (HNPCC). Nonetheless, few studies have examined the influence of the genotype among subjects with a common family history of colorectal cancer (e.g., one or two affected first-degree relatives), and few have assessed potential interactions between dietary and other environmental risk factors and *CCND1* genotype.

We therefore examined the influence of the *CCND1* A870G polymorphism on the risk of colorectal cancer and adenoma in three large prospective cohort studies, the Nurses’ Health Study (NHS), the Health Professionals’ Follow-Up Study (HPFS), and the Physicians’ Health Study (PHS). Using banked blood specimens and regularly updated data on dietary and lifestyle risk factors, we assessed whether the influence of these environmental factors was modified by the *CCND1* polymorphism.

## MATERIALS AND METHODS

### Study subjects

The NHS is an ongoing prospective study of 121 700 US female registered nurses. Details of the design and follow-up of this cohort have been described previously (Chen *et al*, 1998). Briefly, at enrollment in 1976, the participants, who were 30–55 years old, completed a mailed questionnaire providing information on risk factors for cancer and cardiovascular disease. Biennially, updated exposure and disease information is collected by mail, including reports of endoscopy and polyp diagnosis. Self-reported diagnoses of colorectal cancer and adenoma are confirmed through histopathological reports reviewed by a study investigator. From 1989 to 1990, blood samples were collected from 32 826 of the NHS participants. For the analysis of cancers, we confirmed 197 cases of colorectal cancer diagnosed after blood collection through 1 June 2000 among eligible women. In all, 590 women who were free from cancer at the time of case assessment were selected as controls for the cancer cases and matched on year of birth and month of blood draw.

Eligible women for the selection of either an adenoma case or adenoma control were those women who reported a sigmoidoscopy and/or colonoscopy after providing a blood sample and were free from inflammatory bowel disease, a polyposis syndrome, or diagnosed cancer (except nonmelanoma skin cancer). As in previous studies (Chan *et al*, 2004), to avoid detection bias, we defined adenoma cases as one or more pathology-verified adenoma less than 60 cm from the anus. In both the NHS and HPFS, although we asked participants if they underwent sigmoidoscopy or colonoscopy, we did not ask them to specify the type of procedure. Thus, during the study period, for the analysis of adenoma in both cohorts, we assumed that a substantial portion of all procedures were sigmoidoscopies, which encompass only examination of the distal colon and rectum. One control was matched to each case according to year of birth, month of blood draw, fasting status, time period of endoscopy (within 2 years), and routine screening, gastrointestinal symptoms, or family history of colorectal cancer as indication(s) for endoscopy. A total of 557 cases and 557 matched controls were identified. Subsequently, one colon polyp case was identified as hyperplastic and removed from the final analysis, which included 556 cases and 557 controls.

The HPFS began in 1986 when 51 529 US male dentists, optometrists, osteopaths, podiatrists, pharmacists, and veterinarians, aged 40–75 years, responded to a mailed questionnaire (Giovannucci *et al*, 1993). These men provided baseline information on age, marital status, height and weight, ancestry, medications, smoking history, medical history, physical activity, and diet. Exposure and medical history information are updated every 2 years. When a participant reported a diagnosis of colorectal adenoma on the follow-up questionnaires, we asked for permission to acquire the relevant medical records. All cases in this analysis were confirmed through histopathologic reports reviewed by a study investigator. Blood samples were collected between 1993 and 1994 from 18 025 participants, among whom 367 had been diagnosed with adenomatous polyps between 1986 and 1994 and were confirmed by medical records. To be eligible for selection as a case or control, a person must have completed a valid dietary questionnaire in 1986, supplied a blood sample, and have undergone sigmoidoscopy or colonoscopy after 1986, and not have had a cancer diagnosis, excluding nonmelanoma skin cancer, before the date of endoscopy. A total of 736 men who were free from diagnosed polyps at the time of case assessment were selected as controls and matched to each case on year of birth, whether they had had a previous endoscopy, and year of endoscopy. Subsequently, nine controls were identified as cases, so the final set included 376 cases and 725 controls. For the analysis of cancers, we confirmed 182 cases of colorectal cancer diagnosed after blood collection through 1 June 2002 among eligible men. In total, 364 men who were free from cancer at the time of case assessment were selected as controls for the cancer cases and matched on year of birth and month of blood draw.

The PHS is a randomised, double-blind trial of aspirin and *β*-carotene among 22 071 predominantly Caucasian-American male physicians, 40–84 years of age in 1982. Blood samples were collected at baseline, in 1982, from 14 916 (68%) of the randomised physicians. The men were subsequently followed for incident cancer through annual mailed questionnaires. By the year 2000, 272 cases of colorectal cancer were identified and confirmed using medical records. Men who were free from diagnosed cancer at the time of cancer ascertainment (*n*=456) were selected as controls, and were matched on age (±1 year; up to ±5 years for older men) and on smoking history at baseline (current, former, and never-smokers).

### Sample collection

In both the NHS and HPFS, venous blood samples were separated into plasma, buffy coat and red blood cells and stored in liquid nitrogen. Genomic DNA was extracted from whole blood samples (PHS) or from 50 *μ*l buffy coat diluted with 150 *μ*l of phosphate-buffered saline and using the QIAmp (Qiagen Inc., Chatsworth, CA, USA) 96-spin blood protocol according to the manufacturer's instructions. Genomic DNA concentrations were calculated in 96-well format using PicoGreen technology (Molecular Probes, Eugene, OR, USA).

### Cyclin D genotype

Genotyping of *CCND1* was carried out using the TaqMan allelic discrimination system (Applied Biosystems, Foster City, CA, USA). TaqMan primers and probes are available upon request from the authors. Following polymerase chain reaction amplification, end point fluorescence was read with the Applied Biosystems 7900HT instrument and genotypes were assigned using Allelic Discrimination Software (Applied Biosystems SDS Software v.1.7a). Quality control (QC, 10%) samples were included and each analysis included no DNA template controls. Laboratory personnel were blinded to QC and case–control status. In all, 1697 women from the NHS (524 colorectal adenoma cases and 517 matched controls as well as 181 colorectal cancer cases and 475 matched controls) were successfully genotyped, as well as 2275 men from the HPFS (372 colorectal adenoma cases and 712 matched controls as well as 171 colorectal cancer cases and 347 matched controls) and from the PHS (258 colorectal cancer cases and matched controls).

### Statistical analyses

Adenoma risk was considered in relation to the *CCND1* genotypes. *Cyclin D1* A870G was categorised into the three genotypes (G/G, G/A, and A/A) and as noncarriers (i.e., the wild type, G/G) *vs* carriers of the variant A allele (A/A, G/A+A/A). We conducted analyses for all three cohorts separately and then combined analyses, pooling data from the NHS, HPFS, and PHS, with adjustment for age, gender, and body mass index (BMI). For analysis of the main effect of genotype, logistic regression was used to compute odds ratio (OR) and 95% confidence intervals (CIs), control for a variety of potentially confounding variables, and to test for gene–environment interactions. Although the data sets were initially based on matched cases and controls, the stratified analyses required unconditional analysis. Moreover, conditional logistic regression yielded similar results as obtained when running unconditional logistic regression models, thus we report OR) derived from unconditional regression models throughout the manuscript. Specifically, analyses involving NHS, HPFS, and PHS cancer cases and controls were controlled for age, family history of colorectal cancer (NHS and HPFS only), smoking history, aspirin use, BMI, postmenopausal status and postmenopausal hormone (PMH) use (NHS only), physical activity, and intake of red meat, folic acid and alcohol, and history of previous endoscopy (HPFS only). For colorectal adenoma, separate analyses for men and women using unconditional logistic regression were controlled for age, history of previous endoscopy, year of endoscopy, family history of colorectal cancer, smoking history, aspirin use, BMI, PMH use among postmenopausal women (NHS only), physical activity, and intake of red meat, charred meat, folate, and alcohol. In subanalyses, men and women in the adenoma groups were combined for gene–environment interactions and were controlled for age, sex, and family history of colorectal cancer. Analyses involving PHS cancer cases and controls were controlled for age, smoking status, aspirin use, BMI, physical activity, and intake of red meat, vitamins, and alcohol.

The effects of various dietary and lifestyle exposures on colon cancer and adenoma risk were tested in conjunction with *CCND1* genotype. In the *NHS colorectal cancer* group, *CCND1* genotypes were analysed in combination with micrograms (*μ*g) per day of folate including supplements (⩽310, >310), milligrams (mg) per day of vitamin B6 pyridoxine (⩽2.5, >2.5) and grams (g) per day of methionine (⩽1.84, >1.84), based on median distributions in the control population, regular aspirin intake (yes, no), family history of colorectal cancer (yes, no), pack-years of smoking before age 30 (0, ⩽10, >10), g per day of alcohol (<30, ⩾30), age at diagnosis (⩽65, >65), and PMH use among postmenopausal women (never/past, current). In subanalyses, we stratified on the stage of disease (stage I (muscle only) *vs* stages II and III combined (pericolic or perirectal tissue, with or without involvement of lymph nodes)) and site (colon *vs* rectum). In the *NHS adenoma* group, *CCND1* genotypes were analysed in combination with *μ*g per day of folate including supplements (⩽310, >310), mg per day of vitamin B6 (pyridoxine), and g per day of methionine (⩽1.84, >1.84), based on median distributions in the control population, family history of colorectal cancer (yes, no) both alone and in conjunction with adenoma size (small/large), pack-years of smoking (0 years, <25 years, ⩾25 years), g per day of alcohol (<30, ⩾30), age at diagnosis (⩽60, >60), regular aspirin intake (yes, no), PMH use among postmenopausal women (never/past, current), site (proximal *vs* distal), and size (large *vs* small) of adenoma. In the *HPFS adenoma* group, *CCND1* genotypes were analysed in combination with *μ*g per day of folate including supplements (⩽338, 339–496, ⩾497), and g per day of methionine (⩽2.15, >2.15), based on median distributions in the control population, regular aspirin intake (yes, no), age at diagnosis (⩽60, >60), pack-years of smoking (0 years, <25 years, ⩾25 years), g per day of alcohol (<5, 5–30, >30), and family history of colorectal cancer (yes, no). In subanalyses, we stratified on age at diagnosis (⩽60, >60) and site (proximal *vs* distal), and size (large *vs* small) of adenoma. In the *HPFS cancer* group, *CCND1* genotypes were analysed in combination with *μ*g per day of folate including supplements (⩽338, 339–496, ⩾497), and g per day of methionine (⩽2.15, >2.15), based on median distributions in the control population, regular aspirin intake (yes, no), age at diagnosis (⩽60, >60), pack-years of smoking (0 years, <25 years, ⩾25 years), g per day of alcohol (<5, 5–30, >30), and family history of colorectal cancer (yes, no). In subanalyses, we stratified on the stage of disease (advanced *vs* not) and site (colon *vs* rectum). In the *PHS colorectal cancer* group, *CCND1* genotypes were analysed in combination with smoking (never, past, current), vitamin intake (never, past, current), aspirin assignment (yes, no), age at diagnosis (⩽55, >55), site (rectum *vs* colon), alcohol intake in drinks per day (1, ⩾1), and age (⩽55, >55). Exposure information for the NHS was updated from 1990, 1992, 1994, 1996 to 1998. Exposure information for the HPFS and PHS was collected at baseline in 1986 and 1982, respectively.

Statistical tests for interaction was based on the Wald test for the crossproduct term in a model containing the main effects of genotype and exposure variable as continuous variables. All *P*-values are two-sided. All statistical analyses were carried out using the SAS 8.2 statistical package (SAS Institute, Cary, NC, USA).

## RESULTS

### Dietary and lifestyle factors

A recent case–control study (Tranah *et al*, 2005) examined whether the risk factors for colorectal adenoma and cancer were similar in the cases and controls that provided blood samples compared to previous observations for each cohort. For all cohorts, the risk patterns observed for adenoma and cancer cases in this sample were largely similar to those reported for the entire cohort, as reported previously (Giovannucci *et al*, 1993; Giovannucci *et al*, 2003, 1994a, 1994b). Only for the PHS-nested case–control study, the risk factors analysed were not associated with an increased risk of colorectal cancer.

### *CCND1* polymorphism

The *CCND1* A allele frequency ranged from 53 to 56% in the NHS, HPFS, and PHS control populations and the genotype distributions were in Hardy–Weinberg equilibrium. The A allele frequency was slightly more frequent in our populations than in similar, white populations of previous reports (42–43%) (Bala and Peltomaki, 2001; Le Marchand *et al*, 2003; Lewis *et al*, 2003).

In all cohorts combined, the *CCND1* polymorphism did not show statistically significant associations to risk of colorectal cancer (odds ratio (OR), 1.04; 95% CI, 0.82–1.32) or adenoma (OR, 0.96; 95% CI, 0.77–1.18). Moreover, the risk associated with the homozygous AA genotype did not appear to differ materially from the heterozygous GA genotype (Table 1). The *CCND1* A870G polymorphism appeared to confer a modest, although statistically insignificant, increase in the risk of colorectal cancer in women (OR, 1.59; 95% CI, 0.98–2.57). In contrast, the variant genotype was not associated with risk of cancer or adenoma in men or risk of adenoma in women. In addition, *CCND1* genotype did not confer a significant elevation in the risk for either large (⩾1 cm) *vs* small (<1 cm) adenoma in either cohort (data not shown).

We assessed whether the influence of *CCND1* genotype varied for proximal as compared to distal colorectal cancers. The *CCND1* A870G polymorphism appeared to confer an elevated risk for cancers in the distal colon or rectum (rectum to splenic flexure) (OR, 2.84; 95% CI, 1.27–6.33), but not for proximal cancers (splenic flexure to caecum) (OR, 1.26; 95% CI, 0.61–2.60) in the women. In contrast, among men in the HPFS, we found similar risks for both proximal (OR, 1.09; 95% CI, 0.54–2.19) and distal cancers (OR, 0.67; 95% CI, 0.37–1.21). Data on cancer location within the bowel were not available from the PHS cohort. Previous studies reported a younger age of onset for colorectal cancer for subjects with HNPCC who possessed the AA or GA *CCND1* genotype or the presence of the variant truncated *CCND1* transcript (preferentially encoded by the A870G genotype) (Kong *et al*, 2000; Bala and Peltomaki, 2001). We therefore examined whether *CCND1* genotype modified the influence of a common family history of colorectal cancer (i.e., a history in one or more first-degree relatives) on cancer and adenoma risk (Table 2). Among female participants with the GG genotype, a family history of colorectal cancer in one or more first-degree relatives was not associated with a statistically significant elevation in colorectal cancer risk. In contrast, for participants with the GA or AA genotype, a family history was associated with a statistically significant risk of colorectal cancer (multivariate OR, 1.95, 95% CI, 1.13–3.34). After adjusting for other covariates, the influence of genotype on the relation between family history and cancer risk was at the border of statistical significance (*P* for interaction=0.06). However, in this cohort of women, we found no material interaction between genotype, family history and adenoma risk.

Similarly, among those with a family history, there was an increased risk from the GA or AA genotype, compared to those with the wild type. Among men in the HPFS, the influence of *CCND1* genotype on the relation between family history and adenoma risk in the HPFS was statistically significant (*P* for interaction=0.02). Whereas family history conferred no significant influence on adenoma risk among men with the GG genotype, family history was associated with a significantly increased adenoma risk (multivariate OR, 2.28, 95% CI, 1.32–3.95) among men with the GA or AA genotypes. Nonetheless, among men in the HPFS, we found no significant interaction between genotype, family history, and colorectal cancer risk (data on family history were not available from the PHS cohort). In addition, we examined whether age at onset of disease influenced the overall association between the genotype and colorectal cancer or adenomas. In these analyses, we observed a higher risk of colorectal cancer among women who carried one or two copies of the A allele and who had a diagnosis of colorectal cancer at a later age (i.e., at or above age 65 years, as opposed to before age 65 years; AA+AG allele; RR, 2.33; 95% CI, 1.02–5.28). Similar risk increases were not observed for colorectal adenomas among women, or among men.

We also assessed the associations of several dietary and lifestyle factors with risk of colorectal cancer or adenoma stratified by *CCND1* genotype. In analyses of both cancer and adenoma, the influence of BMI, smoking, aspirin use, and intakes of folate, red meat, and alcohol did not differ significantly according to *CCND1* genotype (in the PHS, folate intake was not assessed). However, among postmenopausal women, the inverse relation between postmenopausal oestrogen use and colorectal cancer and adenoma risk appeared to be limited to the A870G polymorphism (Table 3). Among women with the GG genotype, current PMH use did not significantly influence the risk of colorectal cancer or adenoma. However, among women with the GA or AA genotype, current postmenopausal oestrogen use was associated with a significant reduction in the risk of colorectal cancer when compared to never or former users (multivariate OR, 0.57, 95% CI, 0.35–0.92), although a test for statistical interaction was not significant. Similarly, among women with the GA or AA genotype, current postmenopausal oestrogen use was associated with a significant reduction in adenoma risk when compared to never or former users (multivariate OR, 0.63, 95% CI, 0.45–0.87), although the test for interaction was not significant.

## DISCUSSION

In the present study, the GA or AA *CCND1* genotype was associated with a borderline significant increase in the risk of colorectal cancer only among women. In contrast, we found no association between *CCND1* genotype and the risk of distal colorectal adenoma in either women or men. Notably, among postmenopausal women in the NHS, a reduced risk of colorectal cancer or adenoma associated with current oestrogen use was restricted to women with the GA or AA *CCND1* genotype. Postmenopausal oestrogen conferred no benefit among women with GG genotype.

Our findings for colorectal cancer among women are consistent with those of Le Marchand *et al* and others, which found a similar increase in the risk of colorectal cancer among participants with the GA or AA genotype (Kong *et al*, 2000; Porter *et al*, 2002; Le Marchand *et al*, 2003). In contrast to a previous study of colorectal adenoma (Lewis *et al*, 2003), we did not find an association between *CCND1* genotype and the risk of adenoma, although our analysis was limited to adenoma of the distal colon and rectum.

Previous studies suggest that the GA or AA genotype is associated with a younger age of onset for HNPCC. One of these studies found that patients with HNPCC who carried the GA or AA genotype developed colorectal cancer on average 11 years earlier than those with the wild type (Kong *et al*, 2000). However, we are unaware of any studies that have examined the interaction with a sporadic (nonsyndromic) family history. In our study, the presence of at least one A allele substantially augmented the effect of a family history of colorectal cancer on the risk of colorectal cancer in the NHS and the risk of colorectal adenoma in the HPFS. However, we did not find similar interactions for colorectal adenoma in the NHS and colorectal cancer in the HPFS. Given that there was some residual risk for colorectal neoplasia among individuals with the GG group, it appears unlikely that the G-to-A polymorphism is one of the main components and main underlying mechanisms for some nonsyndromic cases of familial colorectal cancer.

Numerous studies observed a significant reduction in the risk of colorectal cancer and adenoma among PMH users (Serrano *et al*, 2004), including a previous analysis of our female cohort (Grodstein *et al*, 1998) and a large randomised trial of postmenopausal women (Chlebowski *et al*, 2004). Nonetheless, no study, to date, has examined whether *CCND1* genotype modifies the influence of PMH use on colorectal cancer and adenoma risk. We found that the benefit associated with PMH use was restricted to those with GA or AA genotype. In the Shanghai Women's Breast Cancer Study, the increased risk of breast cancer associated with oestrogens was limited to women with the GA or AA genotype (OR=2.6) (Shu *et al*, 2005). In contrast, women with the GG genotype experienced no increased risk for breast cancer in association with PMH use (OR=0.8). In a second study, oral contraceptive use was associated with an increased risk of breast cancers with *CCND1* overexpression, but was unrelated to those without *CCND1* overexpression (Terry *et al*, 2002). These data suggest that the *CCND1* GA or AA genotype may augment the various influences of oestrogen in decreasing the risk of colorectal cancer and adenoma as well as increasing the risk of breast cancer. In previous studies, *CCND1* can bind directly to the oestrogen receptor, transactivate oestrogen response elements (Zhou *et al*, 2001), and regulate oestrogen-dependent enhancer activity (Zafonte *et al*, 2000).

The strengths of our study include its relatively large size, prospective design, detailed data on potential confounders, and high follow-up rate. In particular, we had a unique ability to examine a wide range of exposures and interaction with genotype. As participants were health professionals, the accuracy of self-reported data is likely to be high; information on many exposures, including endoscopy, has been validated previously (Willett *et al*, 1985; Rimm *et al*, 1990; Wolf *et al*, 1994). Moreover, because our study was nested within three larger, well-defined cohorts, control participants were sampled from the same population as case participants. Thus, our results are unlikely to be influenced by population stratification or selection bias. Another strength of our study is that we were able to look at both cancer and adenoma as end points. Moreover, our cancer cases were incident cases, which may be important if cyclin D affects survival, as suggested by Le Marchand *et al* (2003); a study which collected DNA after diagnosis may not be able to include fatal cancers.

Our study has several limitations. Firstly, we did not specify on our questionnaire, whether study participants underwent a colonoscopy or sigmoidoscopy. Thus, with regard to our analysis of adenoma, it is possible that some controls were not entirely free of proximal adenomatous polyps (that were beyond the reach of the flexible sigmoidoscope), thus potentially limiting the generalisability of our adenoma findings. Moreover, since we limited our analysis to adenoma within the reach of the flexible sigmoidoscope, we were unable to assess the association of genotype with the risk of proximal adenoma. However, for the analysis of colorectal cancer in our cohort of women, the risk associated with A870G genotype was, in fact, greater for cancers of the distal colon and rectum than for more proximal lesions. Finally, even though our study is of fairly large size, it may still have been underpowered for certain gene–environment interactions.

In summary, our results suggest that the risk of both colorectal cancer and adenoma associated with a family history of colorectal cancer may be augmented by the *CCND1* A870G genotype. Moreover, the A870G genotype appears to enhance the protective effect of postmenopausal oestrogen use on the development of colorectal neoplasia. Additional studies are warranted to confirm this potential interaction between *CCND1* genotype and postmenopausal oestrogen use.

## Figures and Tables

**Table 1:** Associations between *CCND1* A870G genotype and colorectal carcinoma and adenoma risk in the NHS, HPFS, and PHS

***CCND1* G to A**	**Cases (%)**	**Controls (%)**	**OR (95% CI)**
*Cancer overall*			
N	610	1237	
GG	125 (20.5)	264 (21.3)	1.0^a^
AG	311 (51)	593 (48)	1.03 (0.78–1.36)
AA	174 (28.5)	380 (30.7)	1.12 (0.90–1.41)
AA+AG	485 (79.5)	973 (78.7)	1.04 (0.82–1.32)
*P* for trend			0.64
			
*Adenoma overall*			
N	896	1229	
GG	187 (20.9)	241 (19.6)	1.0^a^
AG	441 (49.2)	614 (50)	0.95 (0.77–1.18)
AA	268 (29.9)	374 (30.4)	1.00 (0.78–1.28)
AA+AG	709 (79.1)	988 (80.4)	0.96 (0.79–1.18)
*P* for trend			0.99
			
*NHS cancer* b			
N	181	475	
GG	29 (16)	110 (23.2)	1.0^a^
AG	95 (52.5)	224 (47.2)	1.61 (0.96–2.68)
AA	57 (31.5)	141 (29.7)	1.55 (0.90–2.69)
AA+AG	152 (84)	365 (76.8)	1.59 (0.98–2.57)
*P* for trend			0.16
			
*NHS adenoma* c			
N	524	517	
GG	108 (20.6)	100 (19.3)	1.0^a^
AG	265 (50.6)	266 (51.5)	0.95 (0.67–1.33)
AA	151 (28.8)	151 (29.2)	0.92 (0.63–1.34)
AA+AG	416 (79.4)	417 (80.7)	0.94 (0.68–1.29)
*P* for trend			0.66
			
*PHS cancer* c			
N	258	415	
GG	56 (21.7)	85 (20.5)	1.0^a^
AG	136 (52.7)	203 (48.9)	1.02 (0.68–1.55)
AA	66 (25.6)	127 (30.6)	0.80 (0.51–1.27)
AA+AG	202 (78.3)	330 (79.5)	0.94 (0.64–1.39)
			
*HPFS cancer* b			
N	171	347	
GG	40 (23.4)	69 (19.9)	1.0^a^
AG	80 (46.8)	166 (47.8)	0.86 (0.53–1.40)
AA	51 (29.8)	112 (32.3)	0.81 (0.48–1.38)
AA+AG	131 (76.6)	278 (80.1)	0.84 (0.53–1.33)
*P* for trend			0.45
			
*HPFS adenoma* c			
N	372	712	
GG	79 (21.2)	141 (19.8)	1.0^a^
AG	176 (47.3)	348 (48.9)	0.96 (0.68–1.36)
AA	117 (31.5)	223 (31.3)	0.91 (0.63–1.33)
AA+AG	293 (78.8)	571 (80.2)	0.94 (0.68–1.31)
*P* for trend			0.61

*CCND1*=cyclin D1; CI=confidence interval; HPFS=Health Professionals’ Follow-Up Study; NHS=Nurses’ Health Study; OR=odds ratio; PHS=Physicians’ Health Study.

a Reference group.

b Unconditional logistic regression adjusted for age, family history of colorectal cancer, smoking history, aspirin use, BMI, PMH use, physical activity, and intake of red meat, charred meat, folate, and alcohol.

c Unconditional logistic regression adjusted for age, previous endoscopy, year of endoscopy, family history of colorectal cancer, pack-years smoking, aspirin use, BMI, PMH use (NHS only), physical activity, and intake of red meat, charred meat, folate, and alcohol.

**Table 2:** Relationship between family history of colorectal cancer and risk of colorectal cancer and adenoma among postmenopausal women in the NHS and men in the HPFS, stratified by *CCND1* A870G genotype

	**Family history of colorectal cancer**				
	**No**		**Yes**		
	**Case/control**	**OR (reference)**	**Case/control**	**OR (95% CI)**	***P* for interaction**
*NHS cancer* a *					0.06
GG	22/89	1.0	7/21	1.15 (0.38–3.50)	
GA/AA	113/316	1.0	31/45	1.95 (1.13–3.34)	
					
*NHS adenoma* b					0.77
GG	75/76	1.0	33/24	1.77 (0.84–3.72)	
GA/AA	295/338	1.0	121/79	1.85 (1.31–2.61)	
					
*HPFS cancer* a					0.80
GG	27/59	1.0	13/10	3.15 (1.14–8.67)	
GA/AA	107/241	1.0	24/37	1.44 (0.80–2.57)	
					
*HPFS adenoma* b					0.02
GG	74/126	1.0	5/15	0.52 (0.17–1.60)	
GA/AA	262/543	1.0	31/28	2.28 (1.32–3.95)	

*CCND1*=cyclin D1; CI=confidence interval; HPFS=Health Professionals’ Follow-Up Study; NHS=Nurses’ Health Study; OR=odds ratio.

* Case and control numbers differ from total because of missing information on family history.

a Unconditional logistic regression adjusted for age, PMH use (NHS only), smoking history, aspirin use, BMI, physical activity, and intake of red meat, charred meat, folate, and alcohol.

b Unconditional logistic regression adjusted for age, history of previous endoscopy, year of endoscopy, PMH use, aspirin use, BMI, physical activity, and intake of red meat, well-done meat, folic acid, alcohol, total calories, and caloric-adjusted total fat.

**Table 3:** Relationship between PMH use and risk of colorectal cancer and adenoma among postmenopausal women in the NHS stratified by *CCND1* A870G genotype

	**Postmenopausal hormone use**				
	**Never–past use**		**Current use**		
	**Case/control**	**OR (reference)**	**Case/control**	**OR (95% CI)**	***P* for interaction**
*NHS cancer* a					0.37
GG	14/69	1.0	13/30	2.34 (0.75–7.31)	
GA/AA	95/181	1.0	38/135	0.57 (0.35–0.92)	
					
*NHS adenoma* b					0.33
GG	56/50	1.0	36/34	1.21 (0.55–2.64)	
GA/AA	209/166	1.0	150/189	0.63 (0.45–0.87)	

*CCND1*=cyclin D1; CI=confidence interval; NHS=Nurses’ Health Study; OR=odds ratio; PMH=postmenopausal hormone.

a Unconditional logistic regression adjusted for age, family history of colorectal cancer, smoking history, aspirin use, BMI, physical activity, and intake of red meat, charred meat, folate, and alcohol.

b Unconditional logistic regression adjusted for age, history of previous endoscopy, year of endoscopy, family history of colorectal cancer, aspirin use, BMI, physical activity, and intake of red meat, well-done meat, folic acid, alcohol, total calories, and caloric-adjusted total fat.
